# Artificial intelligence and digital pathology: Opportunities and implications for immuno-oncology

**DOI:** 10.1016/j.bbcan.2021.188520

**Published:** 2021-02-06

**Authors:** Faranak Sobhani, Ruth Robinson, Azam Hamidinekoo, Ioannis Roxanis, Navita Somaiah, Yinyin Yuan

**Affiliations:** a Division of Molecular Pathology, The Institute of Cancer Research, London, UK; b Centre for Evolution and Cancer, The Institute of Cancer Research, London, UK; c Division of Radiotherapy and Imaging, Institute of Cancer Research, The Royal Marsden NHS Foundation Trust, London, UK; d The Breast Cancer Now Toby Robins Research Centre, The Institute of Cancer Research, London, UK

**Keywords:** Artificial intelligence (AI), Deep learning (DL), Digital pathology (DP), Immuno-oncology (IO)

## Abstract

The field of immuno-oncology has expanded rapidly over the past decade, but key questions remain. How does tumour-immune interaction regulate disease progression? How can we prospectively identify patients who will benefit from immunotherapy? Identifying measurable features of the tumour immune-microenvironment which have prognostic or predictive value will be key to making meaningful gains in these areas. Recent developments in deep learning enable big-data analysis of pathological samples. Digital approaches allow data to be acquired, integrated and analysed far beyond what is possible with conventional techniques, and to do so efficiently and at scale. This has the potential to reshape what can be achieved in terms of volume, precision and reliability of output, enabling data for large cohorts to be summarised and compared. This review examines applications of artificial intelligence (AI) to important questions in immuno-oncology (IO). We discuss general considerations that need to be taken into account before AI can be applied in any clinical setting. We describe AI methods that have been applied to the field of IO to date and present several examples of their use.

## 1. Introduction

The ability to evade immune destruction is a seminal feature of cancer [58]. Agents designed to ramp up the anti-tumour immune response have had therapeutic traction across a range of tumour sites and histologies [116] with some patients experiencing durable disease control. Aside from this, traditional cytotoxic therapies have been shown to mediate some of their anti-tumour effects through immune mechanisms [18]. Clinical success from immunotherapy is far from universal and the majority of unselected patients have a poor objective response. Besides, these agents have a significant toxicity profile [50]. To maximise the clinical gains - and minimise harm - it is essential that we have robust predictive biomarkers that are able to prospectively discriminate between those more or less likely to benefit from IO.

### 1.1. Predictive assays in current use

#### 1.1.1. IHC markers

PD-L1 expression by tumour and/or local immune cells, as assessed by single marker immunohistochemistry is used across a spectrum of solid tumours to select for benefit from immune checkpoint inhibitors. However, its utility as a biomarker is limited by intra-tumoural heterogeneity and dynamic changes in expression. We lack a standardised approach to scoring and significance thresholds. Reliability of scoring is affected by inter-observer variation as well as technical differences between the various assays in use [11].

#### 1.1.2. Genomic tools

Genomic tools including targeted panels to estimate tumour mutational burden are also used to select for likely responders. Tumour mutational burden (TMB) correlates with neoantigen load and has been shown to predict response to IO in lung, bladder and head and neck tumours [23]. Cancers with defective mismatch repair (dMMR) tend to have high TMB as consequence, and IO is therefore of particular benefit in this subgroup. dMMR is most commonly seen in cancers associated with the inherited Lynch syndrome (colorectal, endometrial, small intestine, urothelial, central nervous system and sebaceous gland cancers) and can be detected through the use of antibodies against nuclear MMR proteins, plus or minus PCR to identify microsatellite instability - a downstream manifestation of dMMR [83]. Although both are predictive biomarkers for sensitivity to immune checkpoint blockade, TMB and PDL1 do not necessarily select for the same patients as illustrated by the fact that dual checkpoint blockade for NSCLC was beneficial with high TMB, irrespective of PDL1 status [60]. This underlines the fact that clinical response to IO is determined by multiple factors. A recent meta- analysis showed that composite biomarkers incorporating PD-L1, TMB and simultaneous quantification of multiple proteins via multiplex IHC/immunofluorescence performed better than either PD-L1 or TMB in isolation [82]. However, the increased cost and complexity of these techniques need to be considered if aiming to implement more widely.

#### 1.1.3. Assays of immune reaction

The density of tumour-infiltrating immune effector cells also shows promise as a clinically useful biomarker. In colorectal cancer, the Immunoscore has been shown to be a better predictor of outcome than traditional TNM staging. This score is based on the density of CD3 and CD8-positive cells at the invasive margin and the centre of the tumour. Notably, patients who experienced disease relapse had low immune reaction irrespective of the T stage of the primary tumour [90]. A standardised system exists for the manual scoring of stromal tumour infiltrating lymphocytes (TILs) on H&E slides in breast cancer [106]. The score is a semi-quantitative assessment, expressed as an average across all assessable tumour stroma. The intensity of the baseline immune infiltrate has prognostic and predictive significance in HER2- positive and triple-negative subtypes [37]. In triple-negative breast cancer, TILs score predicts pathological and clinical response to checkpoint inhibitors in the neoadjuvant and metastatic settings respectively [21]. Predictive power may be further increased by combining TILs scores with PD-L1 assessment [51]. The consensus TILs scoring methodology represents a pragmatic approach that has shown good rates of inter-user reproducibility. However, its granularity is limited and it does not attempt to capture detail about how immune cells may be distributed within a specimen. Additionally, even a straightforward manual scoring system is time-consuming to implement at scale, for example to analyse a trial cohort with thousands of samples.

### 1.2. Opportunities

A host of clinical trials are currently evaluating novel IO therapies and treatment combinations [116]. Longitudinal tissue specimens collected from patients undergoing treatment with IO are a valuable source of potential information. Studying changes in the distribution and activity of immune cells with therapeutic intervention and correlating these with clinical outcomes can provide mechanistic insights into treatment resistance and identify candidates for predictive biomarkers. In particular, pathological analyses have the advantage of using material such as H&E stained tissue sections, which are widely available and retain information around tissue architecture and spatial organisation. Direct visual assessment of a prepared glass slide using a microscope remains the gold standard in the pathological assessment. However, these traditional manual methods are time-consuming and require a highly trained workforce, which is already under pressure from increasing volume and complexity of histopathology requests [10]. Use of minimally invasive procedures has expanded at the same time as our interest in tissue biomarkers. Therefore pathologists are being asked to report on ever more complex continuous variables, but with less available tissue. Even for an experienced practitioner, manual techniques are inherently vulnerable to inter-and intra-observer variability. There are natural upper limits on precision and limited scope to describe complex topographical features in an objective and quantifiable manner. Digital approaches offer a potential solution to these issues.

## 2. Digital pathology and AI: General principles

In digital pathology (DP), glass-mounted specimens are captured as a whole-slide image (WSI) for downstream computer-based analysis. AI techniques applied to the digitised specimen can utilise various features to perform segmentation and classification tasks. By far the most common AI technique used in these papers and IO research to date is supervised classification. Classification is the task of predicting an output label for each input data point.

**Supervised** refers to the fact that the training model is shown example pairs of inputs and labels, and thereby learns the relationship between the two. The model attempts to draw boundaries – implicitly or explicitly – in the input space, separating data points which belong to different classes. Whilst being considerably easier to train than unsupervised techniques, the drawback of supervised methods is their reliance upon the input of large amounts of labelled ‘ground truth’ data – information collected from the real world, for example, annotations by a pathologist. However it is worth noting that considerable amounts of annotated data are already in existence within the public domain as well as open-source models and easy-to-use software packages.

**Unsupervised** methods, on the other hand, usually bypass the need for labelled data [25,78,79,100,136]. Instead, they rely upon the machine being able to discover relevant features for tasks, such as grouping together unlabelled data points with high similarity. There are four major types of unsupervised methods [49]: (i) exclusive (ii) agglomerative (iii) overlapping and (iv) probabilistic. These models discover unknown patterns in the data, however, in the main, they remain experimental and computationally complex. In specific problems, it can be difficult for the network to converge on a globally optimal solution due to redundant feature representations [24] and it is likely to perform less well than supervised training approaches [144]. However, such methods may be the best approach for truly novel insights. Machine learning (ML) techniques involve a diverse set of models and algorithms but all centre around the concept that computers can learn from data as humans learn from experience, and can make decisions about novel data without the need for ongoing instruction. Of particular interest in our setting are deep learning (DL) models. These consist of cascades of trainable, multi-stage layers inspired by the organisation of neurons. A signal input into the model is propagated and modified in a layer-by- layer fashion along these networks to produce an output. DL models have a wide range of architectures themselves, the choice of which depends on the particular task being solved; for example, in image analysis convolutional neural networks (CNNs) [72], generative adversarial networks (GANs) [52], fully convolutional neural networks (FCNNs) [81] and recurrent convolutional neural networks (RCNNs) [75] are popular choices.

Histopathological image analysis methods can be broadly categorised into cell-level (identifying/segmenting single cells) or semantic region-based (patch-based; larger extracted patches from whole-slide images, i.e. 512pix × 512pix) analysis. Cell-level analysis methods identify structures known as histologic primitives (e.g. nuclei). These features can be correlated with clinical characteristics, such as response to a specific treatment. Early studies applied DL approaches using small patches of manually selected regions of interest extracted from the slides [98]. For example, object detection can be performed by training a deep CNN on patches centred on the objects of interest such as nuclei. These approaches consider only the information within these size-limited patches, which encompass the object and its immediate neighbourhood, and are mostly suitable for identifying small histologic primitives. Accurate detection of these histologic primitives serves as the basis for a larger number of tasks such as morphological grading, molecular profiling and IO assays. Table 1 gives an overview of small size level analysis approaches.

The semantic region-based analysis seeks certain special regions inside the whole section like glands, tubules, ducts, etc. These methods are most suitable for identifying meaningful connectives inside an image. Cell level analysis classifies the patches (often small, i.e. 56 × 56 pixels) of an image into different defined classes while semantic region-based analysis can be regarded as semantic identification of objects in a larger image (i.e. 512 × 512 pixels) in which a pixel-level classification has resulted, i.e. it classifies the pixels into its corresponding classes. Both approaches (cell-level/semantic region-based methods) can be used for different tasks including segmentation, detection and classification based on the type of annotation and ground truth being used in the methodology set-up. Table 2 gives an overview of region-based analysis approaches.

Many reviews of digital analysis of histopathological images exist in the literature and address the various problems associated with the use of different types of histopathology images [17,39,54,56,62,71,93,97,131]. In their recent review Schmauch et al [109] have described numerous recent examples of the applications of AI in oncology and highlight resources and datasets that can help utilise AI tools in cancer research. Table 3 gives an overview of the variety of problems being tackled with DL techniques that are demonstrating promising results.

## 3. Considerations for the use of AI in clinical settings

The backbone of any effective digital pathology service includes (but is not limited to): capturing images using WSI; storing, analysing and archiving the digital images; performing quality control checks; sharing images with other institutions and integrating outputs into clinical decision making. Regulatory requirements and financial viability need to be considered throughout. Workflows require continuous adaptation to evolving demands. In this review, we focus on three main challenges concerning the application of AI algorithms to DP data: (i) generalizability of the model (ii) explainability of the model (iii) limitations on quantity or quality of the data which can be used by the designed model.

### 3.1. Generalizability

This is a measure of how well the complexity of the model matches the complexity of the data. Problems arise when the model has merely memorised training samples but fails to form a general understanding - a problem known as over-fitting. In this case, the model will perform well with training data but fail to identify relevant information in the novel data. The primary goal, and greatest challenge, for any ML practitioner is for the model to correctly apply what it has learned when unleashed on entirely new data. This is crucial for the deployment of AI in DP across hospitals and laboratories. Tables 4 and 5, gives a summary of recent studies in the IO that have evaluated the generalizability of the AI-based models using a large number of internal and external cases. Generalizability may be improved by (i) adjusting network parameters based on the complexity of target data (the greater the number of parameters, the greater the chance of over-fitting); (ii) using dropout neurons (training multiple possible configurations of a network, then calculating the average of all the corresponding subset network weights, which promotes accumulation of independent learning); (iii) weight regularization (to avoid focusing on certain features in the training data, which leads to a continuous increase of weights); (iv) ensuring similar distribution between the training and the upcoming data when deploying the model; (v) frequent re-training rounds (also called fine- tuning) in order to keep up with the change in cohorts.

### 3.2. Explainability

Also known as interpretability, this refers to how well we understand the factors influencing the model’s decision making. It is crucial that a model is explainable when used for healthcare purposes, in order to ensure that predictions are being made in an ethical, reliable and transparent manner. Inability to detect bias could have potentially dangerous consequences. Traditional ‘bottom-up’ ML approaches focus their analysis on specific fundamental characteristics and micro- attributes of a histology image. Deconvoluting the decision-making processes in this scenario is more intuitive and can be approached in several different ways including activation maps (and its derivatives) [22], as well as attention methods [44] and compensating dataset bias and scarcitys [140].

By contrast, it can be very difficult to identify the salient features being used by the model when using an end-to-end DL approach. For example, Courtiol et al. [36] identified strongly associated features with either progression/survival; however, some of these features were unexpected (i.e. stromal regions with inflammation and other histological features that were not within the tumour microenvironment). However, progress has been made in this area and there are examples in the literature where DL has yielded biologically interpretable results. For example, Beck et al. [14] developed a prognostic model incorporating morphometric descriptors and higher-level contextual image features and implicated stromal morphologic structure as a prognostic determinant for breast cancer. Ali et al. [4] designed spatially aware cell cluster graphs to predicting tumour outcome in Oropharyngeal p16+ and showed that combining stromal and epithelial nuclear architectural contributions yield superior prognostic performances. Yamamoto et al. [136] extracted explainable features from histopathology images and several studies have addressed patient stratification by DL methods using H&E images through identifying specific areas of tissue strongly associated with either progression or survival [80,91,115]. As pathologists will retain overall clinical supervision for conclusions drawn from patient samples, transparency is needed in order for them to understand when algorithms should be applied and under what circumstances the output should be used with caution [61].

### 3.3. Quantity and quality of data

Digital techniques require the pathology specimens to be scanned at high resolution. Investment in infrastructure is required to cope with this additional step in the pre-diagnostic pipeline, and also to store the colossal amounts of data (e.x, one H&E slide with 20× magnification has a file size of 473,869,300 bytes) with appropriate security considerations and inventory management capabilities. The advent of a graphics processing unit (GPU) based processing, in which vast amounts of data is handled in a parallel fashion has enabled up-scaling to extremely large neural networks which allow huge training sets to be loaded and processed. The quality of the acquired digital images needs to be certified and accepted both by pathologists and the Computer-Assisted Diagnosis system. Presence of artefacts or unintentional loss of information during data acquisition can have a significant influence on down-stream processing. Digital image artefacts may be introduced at any point along the pathway of histopathology slide preparation, from surgical removal through to fixation, tissue processing, embedding, microtomy, staining, mounting, as well as the final digitisation step [117]. It is important to be able to identify commonly occurring artefacts such as blurriness, over-straining, air bubbles and colour variation which would adversely affect the interpretation and cause the sample to be diagnostically useless. To address these issues, various preprocessing methods have been proposed to reduce noise: conversion to grayscale, colour normalization [30,32,42,68] or colour augmentation [73,76].

Alternatively, Janowczyk et al. [64] proposed an automated quality control approach to precisely localize artefacts on slides to be avoided during computational analysis. Steiner et al. [117] have developed a novel convolutional neural network (DeepFocus) to automatically identify out-of-focus regions in histopathological images. In addition, results of medical interest such as survival prediction are sensitively influenced by the accuracy of the designed algorithm. Most of these medical approaches are supervised methods therefore require ground truth annotations. For most problems, the expert opinion of histopathologists and other medical doctors provide the gold standard for training automated decision support systems. However, in many settings, it may be impossible for clinicians to provide this training information with absolute certainty. In summary, although the performance of an algorithm is often measured by accuracy this is not the only feature that is required if the tool is to be of use in everyday applications, including in the field of IO. Training a model on diverse and noisy clinical cohorts will cause accuracy to decrease, but is of pivotal importance in achieving a generalizable algorithm. It is crucial that any model undergoes careful and rigorous validation, preferably within the context of a multicentre prospective trial [12]. Once applied in real- world scenarios, a clinical team will still be required to make a final judgement on the utility of the output for any individual, bearing in mind the additional context and influencing factors.

## 4. AI methodology in the field of IO

In Table 4, we present some of the DP approaches that have been used to facilitate different pathology workflows for various immune biomarkers, some of which have characterised the TME through spatial analysis and multiplexing. In Table 5, we present non-comprehensive collections of DP approaches that have been used to facilitate different pathology and data integration workflows for IO. This body of work has characterised the TME through cell analysis, spatial analysis, multiplexing, and omics data integration. The rest of this section discusses four main areas in depth.

### 4.1. Applications in IO research

#### • Evaluating TME topography -

The functionality of individual cells within the TME is influenced by their precise location, including proximity to other cell types and features of the supporting stroma. Macrophages, for example, display location-dependent phenotypic plasticity; behaviour varies according to whether they are located in the invasive, stromal or hypoxic zones of the tumour [138]. Single-cell RNA sequencing has contributed to the discovery of functionally distinct cell subsets in the TME, which hold independent prognostic and predictive value in determining response to immunotherapy [13]. Tissue sections preserve spatial information and are therefore an ideal substrate for computational analysis of topographical patterns. DL-based image analysis has been used extensively to study the spatial organisation of the immune infiltrate across cancer types, revealing rich and diverse patterns from routine clinical H&E [43]. Effland et al. [41] demonstrate the use of an ML algorithm which can detect immune cells in the immediate neighbourhood of tumour cells. The model could also be used to identify immune cells proximate to other immune cells, and thereby define immune-rich zones. One interesting aspect of this work was the use of an artificial training dataset, generated stochastically from a handful of real-life images. This approach avoids the requirement for extensive numbers of annotations by pathologists but may threaten generalizability. Fibroblasts may provide growth factors and extracellular matrix components providing an extrinsic mechanism of immune- escape. Using a combination of flow cytometry and spatial histology assessment, studies in both breast and pancreatic cancer independently identified specific immunosuppressive fibroblast subsets that localize to the boundary of tumour nests [35]. The observations of specific spatial compartmentalization of these cell subsets are intriguing, and automated spatial histology analysis could help accelerate and standardize such studies. For example, Failmezger et al. [43] have recently demonstrated the use of network topological analysis to define a physical barrier of lymphocytic infiltration formed by stromal cells within the TME of metastatic melanoma. In lung cancer, the fractal complexity of the cancer-stromal cell interface has been used to characterise the spatial arrangement of immune cells [1]. The box-counting algorithm, also known as the Minkowski–Bouligand dimension, was modified in order to capture coarse-to-fine geometric details of the cancer-stroma interface over a range of spatial scales determined by cell distributions. Using this method complex morphological patterns dictating cancer-stromal cell contact emerged, which were preserved over varying spatial scales. Fractal dimension was significantly higher in immune- cold tumour regions, and this could not be explained by stromal cell abundance. This supports the conclusion that stroma-based inhibition associated with immune cold phenotypes is a specific morphological pattern. Spatial measures of the immune response such as these have been shown to correlate with resistance to immunotherapy and with patient outcomes, and therefore have the potential for clinical application as predictive biomarkers.

#### • Optimisation of immune scoring -

The availability of AI tools in DP has renewed interests in the development of immune scores for predicting prognosis and response to immunotherapy. Koelzer et al. [69] demonstrated an example of computational quantitation of membranous PDL1 expression using multiplexed IHC and the HALO™ digital image analysis software. The authors then employed a supervised machine learning algorithm (random forest model) to classify and exclude immune cells from analysis. By restricting PD-L1 scoring to melanoma cells, the authors aimed to reduce apparent heterogeneity which would otherwise lead to artificially high scores. The checkpoint inhibitor ipilumimab is an antibody directed against cytotoxic T-lymphocyte antigen (CTLA-4). There is an unmet need for biomarkers predicting response to CTLA blockade. Harder et al. [59] used an AI approach to discover novel immune-based signatures associated with clinical response. WSI were generated from melanoma biopsies taken prior to exposure to ipilumimab, slides had been stained for CD3, CD8, and FoxP. Objects of interest (CD4 and CD8 positive cells) stained in a similar way to melanin and therefore a DL classification step was used to identify the immune cells. Image-based features from regions of interest were then extracted and mined for correlation with patient outcomes, although the small sample size was limiting in this study with respect to clinically translatable conclusions. Successful digital approaches to TILs scoring not only enhance speed and precision but also permit the integration of spatial information [6]. For example, in early-stage lung cancer, a set of spatial descriptors of co-localisation patterns of TILs and tumour cells were associated with recurrence [34]. In bronchoscopic biopsies from pre- invasive lesions, regressive carcinoma-in-situ lesions harbour more infiltrating immune cells, measured by AI and DP, than those that progress to cancer, suggesting that host immune surveillance is strongly implicated in regression of such lesions [95]. Conversely, the presence of a poorly-infiltrated tumour is a negative prognostic indicator in solid tumours. For example, in one of the first studies to investigate the immune landscape across multiple metastases using pathological samples, the immunoscore for the least immune-infiltrated metastases was found to be the strongest prognosticator in colorectal cancer [89]. Similarly, multi-region sampling in lung cancer found a strong association between the number of tumour regions with diminished lymphocytic infiltration and the risk of disease relapse. Prognostic value was independent of tumour size and stage and further validated in an independent cohort of 970 patients with 4324 multi-region tumour samples, representing the largest multi-region fully automated computational pathology analysis to date [1]. Thus, even if there is above-average immune infiltration across the tumour(s) as a whole, it is the presence of immune-cold regions which appears to drive the clinical outcome and is, therefore, the more significant feature. Automated techniques can enhance our ability to detect such regions. Neural networks enable the integration of heterogeneous data. Reiman and colleagues demonstrated a model which incorporated bulk RNA sequencing data and morphological features from H&E specimens to estimate abundance of immune cell subtypes. This enabled the identification of key effector immune cells without the need for more specialised laboratory techniques such as multiplexed immunofluorescence or single-cell RNA sequencing [99]. The approach was flexible and the authors envisioned that additional clinical or molecular information could be incorporated, such as radiological features or data from methylation assays. Thus DP and AI could be applied to the measurement of composite, multi-modality biomarkers.

#### • Accounting for intra-tumoural heterogeneity in biomarker development -

When assessing the immunogenicity of a given tissue sample, pathological and molecular approaches may produce discordant results. Spatial heterogeneity may also account, at least in part, for the lack of reproducibility in molecular testing on diagnostic tumour samples, due to sampling bias. Indeed, up to 50% of patients from a multi- region dataset were vulnerable to this issue when using published prognostic signatures [19]. Identifying genes expressed uniformly (‘clonally’) across different regions within the same tumour, and deriving a molecular read-out on this basis is likely to be more robust to this variable than conventional methods. The ORACLE signature was significantly associated with mortality in a meta-analysis of 904 lung cancer patients sourced from five separate cohorts. In a study using multi-region sampling, DL pathological image analysis and RNA- sequencing data were derived from the same frozen tissue samples in non-small cell lung cancer [1]. Immune assessment based on these two data types were in agreement in the majority of samples, with the exception of patients that exhibited high intra-tumoural heterogeneity of immune cell distribution as based on RNA- and exome-sequencing data. Moreover, in the discordant tumour regions, pathological images showed a high level of spatial heterogeneity in TIL distribution, measured by immune spatial clustering. Thus, spatial heterogeneity of lymphocyte distribution is likely to be the explanatory factor for the discrepancy between data types generated from adjacent tumour sections. Approaches such as this that consider intra-tumoural heterogeneity may help overcome the reproducibility problem for tumour molecular biomarkers.

#### • Deciphering cancer evolution towards immune escape -

The TME can be considered as an ecosystem made up of interacting populations of cancer cells and stroma [86,126]. Intra-tumoural genetic diversity of cancer cells provides a substrate for evolution according to Darwinian principles [53]. The anti-cancer host immune response, enhanced by IO therapeutics, exerts a selective force which favours expansion of clonal populations that are able to resist this pressure – this is known as immunoediting [104]. Immune-escape may be mediated by cancer-cell intrinsic adaptations, such as modulation of immune checkpoint pathways, or through selection advantages conferred by the cancer-associated stroma [38,122]. By combining pathological immune scoring with sequencing efforts, it has been shown that immune edited tumour clones of colorectal cancer were eliminated while progressing clones were immune-privileged, such that branched evolution across space and time could be traced back to immune-escaping clones [7]. In high-grade serous ovarian cancer, a negative association between epithelial CD8+ TILs scored using AI and DP and cancer genetic diversity was found, providing evidence of immunological pruning of tumour clones [141]. Thus, DP coupled with omics data will allow the expanded application of these techniques to discover unique spatial signatures that signify immune regulation and evasion.

## 5. Conclusion

AI and DP tools, tailored for use with routine clinical samples and cutting-edge multiplex tissue imaging techniques have the potential to enable precise descriptions of the complex spatial organisation of the tumour ecosystem to emerge. Integrating this information with genomic and transcriptomic data could unveil mechanisms of immune escape evolving with and without treatment. AI could therefore drive the discovery of novel biomarkers of immune sensitivity and resistance, and identify novel therapeutic targets DL approaches have been popular in early computational pathology efforts. However, there are unavoidable challenges in their application to clinical data. Many current DL algorithms are regarded as ‘black box’ models, for which it is difficult to produce an explanation for a particular predictive outcome or identify the salient features upon which a decision was made. This is one reason why it has not yet yielded validated, comprehensive, high-level systems. A collaborative approach between data scientists and clinical pathologists in this field will provide the optimal conditions for the development of robust solutions that are sufficiently interpretable to cross into clinical use.

## Supplementary Material

1

## Figures and Tables

**Table 1:** Overview of papers using deep learning for digital pathology at cell level for various tasks including detection, segmentation, and classification.

Reference	Topic	Staining	Method
[105]	Mitosis detection	H&E	CNN-based pixel classifier
[84]	Mitosis detection	H&E	combines shape based features with CNN
[123]	Mitosis detection	H&E	CNN and handcrafted features
[110]	Mitosis detection	H&E	CNN-based patch classifier
[120]	Mitosis detection	–H&E	–CNN-based mitosis detection
[26]	Mitosis detection	–H&E	CNN
[3]	Mitosis detection	H&E	fCNN, CNN for segmentation
[85]	Mitosis detection	–	hierarchical CNNs for patch sequence classification
[77]	Mitosis detection	–	survey on nuclei analysis
[62]	Nuclei detection	IHC	review on nuclei detection
[111]	Nuclei detection	H&E	spatially constrained CNN
[129]	Nucleus detection	H&E, Ki-67	CNN-based structured regression model
[128]	Nucleus detection	Ki-67	CNN model
[2]	Cell detection	H&E	CNN
[112]	Nucleus detection	H&E	CNN
[66]	Nucleus detection	H&E	combination of CNN and hand- crafted features
[135]	Nucleus detection	–	general deep learning framework
[127]	Nucleus detection	FL, H&E	fully convolutional regression networks
[102]	Tubule nuclei detection	H&E	CNN-based classification
[119]	Nucleus detection	H&E	CNN-based classification of superpixels
[133]	Nucleus detection	H&E	stacked sparse auto-encoders (SSAE)
[121]	Nuclear area measurement	H&E	CNN
[28]	Nucleus classification	IFL	Deep regression network (DRN)
[57]	Nucleus classification IFL	H&E	CNN
[88]	Classification of mitochondria EM	EM	CNN-based patch classifier
[96]	Nucleus classification FL	H&E	pre-trained CNN
[3]	Nucleus classification	IHC	CNN
[139]	Nucleus classification H&E	H&E	–DNN
[125]	Subtype cell detection	H&E	combination of two CNNs
[132]	Nucleus segmentation	H&E, IHC	CNN and selection-based sparse shape model
[47]	Nucleus classification IFL	IFL	CNN
[142]	Classification of leukocytes RM	RM	CNN-based patch classifier
[114]	Nuclei segmentation	H&E	multi-scale CNN and graph- partitioning-based method
[103]	Cell segmentation	–	U-Net with deformation augmentation
[63]	Nucleus segmentation H&E	H&E	deep hierarchical learning scheme
[2]	Nuclei segmentation	–	extracted bounding box information
[137]	Glial cell segmentation TPM	TPM	fCNN with an iterative k- terminal cut algorithm
[113]	Cell segmentation H&E	H&E	multi-scale CNN
[95]	Cell detection	H&E,IHC	deconvolving convolutional neural network
[55]	Cell detection	H&E,IHC	Concordent
[143]	Tissue classification	H&E	multispectral unsupervised feature learning

**Table 2:** Overview of papers using deep learning at tissue level for various tasks including detection, segmentation, and classification.

Reference	Topic	Staining	Method
[33]	Segmentation of neuronal membranes	EM	Ensemble of several CNNs with different architectures
[65]	Segmentation of colon glands	H&E	Used two CNNs to segment glands
[8]	Detection of lobular structures in breast	IHC	CNN and a texture classification
[15]	Segmentation of colon glands	H&E	fCNN with a loss accounting
[16]	Segmentation of colon glands	H&E	A multi-loss fCNN
[29]	Neuronal membrane, fungus segmentation	EM	Combination of bi- directional LSTM-RNNs and kU-Nets
[27]	Segmentation of colon glands	H&E	deep contour-aware CNN
[31]	Segmentation of xenopus kidney	CM	3D U-Net
[40]	Segmentation of neuronal structures	EM	fCNN with skip connections
[74]	Segmentation of colon glands	H&E	compares CNN with an SVM using hand-crafted features
[124]	Segmentation of messy, muscle regions	H&E	conditional random field jointly trained with an fCNN
[130]	Perimysium segmentation	H&E	2D spatial clockwork RNN
[134]	Segmentation of colon glands	H&E	used three CNNs to predict gland and contour pixels
[128]	Segmenting epithelium & stroma	H&E, IHC	CNNs applied to over- segmented image regions
[48]	Detection and classification of cancer in whole slide breast	H&E	detection, classification and pixel-wise labeling of WSI
[101]	Pixel-wise classification	H&E, IHC	semantic segmentation using a FCN

**Table 3:** Overview of held challenges in the field of digital pathology.

Name	Aims	tissue	Dataset released			Year	Provided ground-truth
			Staining	Training	Testing		
ICPR https://mitos-atypia-14.grand-challenge.org/	mitosis detection, nuclear atypia score	breast	H&E	32 WSIs		2014	centroids of mitosis, nuclear atypia score
GlaS https://warwick.ac.uk/fac/sci/dcs/research/tia/glascontest/	gland segmentation	colon	H&E	85 images	80 images	2015	binary masks
BioImaging http://www.bioimaging2015.ineb.up.pt/challenge_overview.html	ccancer classification	breast	H&E	140 images of 2048×1536	20 images	2015	labels
TUMAC http://tupac.tue-image.nl	tumour detection	breast	H&E	573 WSIs	321 WSIs	2016	tumour proliferation score, molecular proliferation score.
CAMELYON’16 https://camelyon16.grand-challenge.org/	detection of cancer metastasis	breast	H&E	270 WSIs	130 WSIs	2016	annotated contours, binary masks
HER2 Scoring https://warwick.ac.uk/fac/sci/dcs/research/tia/her2contest	HER2 scoring	breast	IHC	100 WSIs		2016	HER2 and %age scores
TMA analysis in thyroid cancer diagnosis http://www-o.ntust.edu.tw/~cvmi/ISBI2017/	cancer diagnosis	thyroid	H&E, IHC	28 TMAs, 616 tissue cores		2017	–
CAMELYON’17 https://camelyon17.grand-challenge.org	detection of cancer metastasis	breast	H&E	1399 WSIs		2017	metastases annotations in WSI, patient pN-stage label
BACH https://iciar2018-challenge.grand-challenge.org/	classification and pixel-wise labelling of WSIs	breast	H&E	400+ images, 10 WSIs	20 WSIs	2018	pixel-wise labels
PatchCamelyon https://patchcamelyon.grand-challenge.org	metastasis detection	lymph node		327,680 images		2018	binary label indicating presence of metastatic tissue
ACDC-LungHP https://acdc-lunghp.grand-challenge.org/	cancer detection, classification	lung	H&E	150 WSIs	50 WSIs	2019	annotation of cancer regions
ANHIR https://anhir.grand-challenge.org/Intro/	image registration	lesions, lung- lobes, mammary- gland	H&E, IHC	50+ WSIs		2019	–
LYSTO https://lysto.grand-challenge.org/LYSTO	assessment of lymphocytes	breast, colon and prostate	IHC	20,000 patches of size 299×299	12,000 patches	2019	number of lymphocytes for each patch
DigestPath https://digestpath2019.grand-challenge.org/Home/		mucus-secreting glands	H&E	99 WSIs	56 WSIs	2019	cell bounding boxes
PAIP https://paip2019.grand-challenge.org/Home/	liver cancer Segmentation	liver	H&E	60 WSIs	40 WSIs	2019	tumour area segmentation, viable tumour area
CodaLab https://competitions.codalab.org/competitions/20395#learn_the_details-overview	classification normal cells	blood	–	73 cases	45 cases	2019	lables
LYON https://lyon19.grand-challenge.org/Home/	lymphocyte detection	breast, colon and prostate	IHC	no training data	441 region of interests	2019	–
ECDP2020 https://ecdp2020.grand-challenge.org/Home/	identify HER2+ from HER2-	breast	H&E	360 WSIs		2020	–
Gleason https://gleason2019.grand-challenge.org	gleason grading	prostate	TMA (H&E)	245 cores	88 cores	2019	maps and labels

**Table 4:** Overview of different pathology workflows for various immune biomarkers that have been addressed by deep learning approaches.

Reference	Aims	Methodology		Dataset used		Results
		Task	Approach	Tissue	Modality	
[119]	quantification of tumour-infiltrating immune cells	supervised classification of immune cell-rich/poor regions	1-features extraction by CNN 2-binary classification by SVM	breast	H&E, CD45	F-score = 0.94; K_DL=0.79_ vs *Kmanual* = *078*
[107]	spatial organisation and molecular correlation of TIL maps with survival, tumour subtypes, and immune profiles	1-supervised classification of patches with low/high lymphocyte by CNN; 2-Supervised segmentation of necrosis regions by CNN	lymphocyte and necrosis semi-supervised CNN	various	H&E, molecular data	–
[87]	quantification of immune infiltrates in situ in the environment of epithelial and stromal compartments	–	–	lung	TMA (including: CD8, CD20, CD4, FOXP3, CD45RO, and pancytokeratin)	correlation of DL vs manual lymphocytes quantification for: CD45RO (*R* = 0.52), FOXP3 (*R* = 0.87), CD4 (*R* = 0.79), CD20 (*R* = 0.81),CD8 (*R* = 0.90)
[118]	patient outcome prediction	supervised classification of samples into low/high digital risk score	1-feature extraction with a deep CNN; 2-feature pooling with IFV; 3-PCA; 4-classification with SVM	breast	TMA	ACC_*automated*_ = 0.60 (95% CI 0.55–0.65) vs ACC_*manual*_ = 0.58 (95% CI 0.53–0.63)
[5]	region and nucleus segmentation for characterisation of TILs	1-supervised classification of histologic compartments; segmentation of nucleus; calculate TIL scores;	1-FCN to output a combined mask. 2-decomposing output for region and nucleus segmentation; 3-seed classifications from the cell segmentation.	breast	H&E	Dice = 0.78, ROC-AUC = 0.89, *R* = 0.73, *p* < 0.001
[9]	quantification of biomarkers of immune cells	supervised binary classification	1-features extraction by a CNN; 2-binary classification by softmax	lung	CD3, CD8, CD20	cell count difference to humans = 0.033 cells on average
[70]	precision immunoprofiling, digital scoring of PD-L1 expression		characterization of the tumour microenvironment through spatial analysis and multiplexing; spatial analysis of T-cell infiltrationn	colon	TMA	–
[20]	checkpoint inhibitor response prediction using patient derived xenografts in humanized mice	tissue classification using HistoNet model with eight distinct classes	automatic extraction of meta-features for the characterisation of the tumour	H&E	lung (mouse-trial)	F1-score of 83%; ACC_*tumor-response*_ = 84%

**Table 5:** Overview of different collections of DP approaches that have been used to facilitate data integration work-flows for IO.

Reference	Topics	Aim	Summary
[108]	A deep learning model to predict RNA-Seq expression of tumour from whole-slide images	Predict RNA-Seq profiles from whole-slide images	The developed model (HE2RNA) could predict subsets of genes expressed in different cancer types and the expression of a subset of proteincoding genes. It could also quantify immune infiltration, including genes involved in immune cell activation status and immune cell signalling
[46]	PanNuke Dataset Extension, Insights and Baselines	Release the PanNuke dataset for nucleus segmentation and classification; eliminate the process of verification and quality control by the clinical professionals.	Comparing instance segmentation performance of several models using the prepared PanNuke dataset. The models trained on PanNuke generalise to other unseen tissues.
[45]	Pan-cancer computational histopathology reveals mutations, tumour composition and prognosis	pan-cancer computational histopathology (PCCHiP) study associations between computational histopathological features and genomic driver alterations, whole transcriptomes and survival within the pan-cancer computational histopathology (PCRCHiP)	Pan-cancer computational histopathology analysis with deep learning extracts histopathological patterns and accurately discriminates 28 cancer and 14 normal tissue types. Computational histopathology predicts wholegenome duplications, focal amplifications and deletions, as well as driver gene mutations
[67]	Pan-cancer image-based detection of clinically actionable genetic alterations	Use deep learning to predict point mutations, molecular tumour subtypes and immune-related gene expression signatures directly from routine histological images of tumour tissue	Deep learning can predict point mutations, molecular tumour subtypes and immune-related gene expression signatures directly from routine histological images of tumour tissue
[91]	Predicting cancer outcomes from histology and genomics using convolutional networks	Developed a computational approach based on DL to predict the overall survival of patients diagnosed with brain tumours from microscopic images of tissue biopsies and genomic biomarkers, present an approach called survival convolutional neural networks (SCNNs), which provide a highly accurate prediction of time-toevent outcomes from histology images	Approach surpasses the prognostic accuracy of human experts using the current clinical standard for classifying brain tumours and presents an innovative approach for the objective, accurate and integrated prediction of patient outcomes.
[92]	Unmasking the tissue microecology of ductal carcinoma in situ with deep learning	Automate the identification of DCIS; quantify the spatial relationship of DCIS with TILs, providing a new way to study immune response and identify new markers of progression improving clinical management	Developed a deep learning pipeline that integrates tissue segmentation, DCIS segmentation, single cell classification and spatial analysis in routine H&E histology images
[94]	An artificial intelligence algorithm for prostate cancer diagnosis in WSI of core needle biopsies: a blinded clinical validation and deployment study	Predict slide-level scores for probability of cancer, Gleason score, Gleason pattern, and perineural invasion and calculation of cancer percentage present in CNB material	The trained model was tested on internal and external datasets elucitating generalizability of the algorithm
